# Gene Expression, Oxidative Stress, and Senescence of Primary Coronary Endothelial Cells Exposed to Postprandial Serum of Healthy Adult and Elderly Volunteers after Oven-Cooked Meat Meals

**DOI:** 10.1155/2017/3868545

**Published:** 2017-12-12

**Authors:** Costarelli Laura, Giacconi Robertina, Francesco Piacenza, Andrea Basso, Deborah Pacetti, Michele Balzano, Riccardo Gagliardi, Natale Giuseppe Frega, Eugenio Mocchegiani, Mauro Provinciali, Marco Malavolta

**Affiliations:** ^1^Advanced Technology Center for Aging Research, Scientific and Technological Pole, Italian National Institute of Health and Science on Aging (INRCA), Ancona, Italy; ^2^Department of Agricultural, Food, and Environmental Sciences, Polytechnic University of Marche, Via Brecce Bianche, 60131 Ancona, Italy; ^3^R&D department, Eureka Lab Division S.r.l., Via E. Fermi, 25, 60033 Chiaravalle, Italy; ^4^Ctr. Nutrition and Aging, Scientific and Technological Pole, Italian National Institute of Health and Science on Aging (INRCA), Ancona, Italy

## Abstract

Epidemiological studies have linked high consumption of meat with major age-related diseases including cardiovascular diseases. Abnormal postprandial increases in plasma lipids after a meat meal have been hypothesized among the pathogenetic mechanisms. However, it is still unknown if the postprandial serum derived after a normal meat meal is able to affect endothelial function, and if the type of meat and the age of the donors are critical factors. Here, we show the effects of postprandial sera derived from healthy adults and elderly volunteers who consumed meat meals on human coronary artery endothelial cell (HCAEC) oxidative stress, gene expression, DNA damage, and cellular senescence. We observed that a single exposure to postprandial serum induces a slight increase in ROS that is associated with modulation of gene expression pathways related to oxidative stress response and metabolism. The postprandial-induced increase in ROS is not associated with a measurable DNA oxidative damage. However, repeated exposure to postprandial serum induces an acceleration of cellular senescence. Taking into account the deleterious role of cellular senescence in age-related vascular diseases, the results suggest a new mechanism by which excessive meat consumption and time spent in postprandial state may affect health status during aging.

## 1. Introduction

Many epidemiological studies have linked high consumption of processed meat, particularly processed red meat with cardiovascular disease (CVD) [1], stroke [2], diabetes [3], and colorectal cancer [4]. This is partly attributed to the generation of oxidation products during thermal processing of meat [5], as well as to the proinflammatory and prooxidative condition of the postprandial state [6]. Postprandial elevated triglycerides have been reported to impair endothelial function [7] and exert proapoptotic effects on endothelial cells [8]. Gene expression profiling of endothelial cells exposed to hyperlipaemic postprandial sera from healthy volunteers after a high-fat challenge showed increased transcription of genes involved in growth arrest and apoptosis [9]. However, it is still unknown if the postprandial serum derived after a normal meat meal is able to affect endothelial function in a way similar to that shown after a high-fat challenge. Exposure of normal human coronary artery endothelial cells (HCAEC) to postprandial sera of healthy volunteers after a meat meal which includes commonly consumed drinks, side dishes, and spices [10] can provide a model to mimic the physiological impact of postprandial state on the endothelial cell [11]. Most importantly, the type and quality of the meat and other components of the meal as well as age and health status of the individual consuming the food can alter the characteristics of the postprandial response during a normal meal [12–14]. Even given that a normal meal may provide a lower physiological impact (e.g., postprandial lipid increase) compared to a fat challenge, the postprandial status may induce subcytotoxic stress that is detrimental on the long run. For example, models of repeated short exposure to subcytotoxic stressors (UV, hyperoxia, hydrogen peroxide, etc.) display cellular senescence as a common fate [15, 16]. Here, we report the effects of postprandial sera derived from healthy adult and elderly volunteers who consumed meals based on various type of meats on HCAEC oxidative stress, DNA damage, and cellular senescence while providing mechanistic insights by gene expression analysis.

## 2. Material and Methods

### 2.1. Study Design

The study group consisted of six adult (age range 26–51 years) and six elderly (age range 66–73 years), healthy normolipemic, nonsmoking males. All volunteers had normal physical examinations without any medical history of digestive, renal, cardiovascular, endocrine, or chronic diseases. The physical characteristics of the adult subjects were (mean ± SD) age (years) 39.3 ± 11.2, body weight (kg) 81.3 ± 4.9, and BMI (kg/m^2^) 24.8 ± 2.4. The physical characteristics of the elderly subjects were (mean ± SD) age (years) 68.7 ± 3.4, body weight (kg) 72.7 ± 6.71, and BMI (kg/m^2^) 22.3 ± 1.4. The purpose and potential risks of the study were explained to all subjects, and their written consent was obtained before participation. The study was carried out after approval by the ethics committee of INRCA.

Volunteers were instructed to consume each Tuesday and Thursday of the week a specific meal from 3 different types: (1) a pork meat- (PM-) based meal, (2) a rabbit meat- (RM-) based meal, and (3) a chicken meal- (CM-) based meal. All the food material was weighted and delivered to the home of each volunteer the day before consumption. Volunteers were also instructed to not change their weekly dietary habits for the whole length of the study (75 days, approximately 10.5 weeks were needed to complete all meals for all subjects) and to follow specific cooking instructions for the experimental meals. The meals were designed to accomplish the common Italian tradition to accompany oven-cooked meats with roasted potatoes and to use condiments such as rosemary, salt, and extra virgin olive oil. The main food material consisted of 200 g (all edible) of pork chop (PM meal) or a 250–350 g (approx. 60%–70% edible) of chicken quarter (CM meal) or 400 g (approx. 50% edible) of rabbit pieces (RM meal) obtained from local producers. The meal included also 5 ml (4 g) of extra virgin olive oil, 200 g of potatoes (carbohydrates 17.5%, protein 2%, and moisture 79%), 0.5 g of rosemary, and 2 g of salt (commercial products from a local market). All condiments were added to the meat and cooked in an oven at 180–200°C for 45–60 min, with the last 5–10 min of grill. The range of temperature and cooking time was established in order to meet the individual preferences of the volunteers. Drinks consisted of water ad libitum and a half glass of red wine (150 ml, 12.5% alcohol). Fresh pork chops showed the following proximate composition (g/100 g meat): protein 19.2, total fat 11.4, moisture 68.0, and ash 1.4. Rabbit pieces showed the following proximate composition: protein 22.0, total fat 2.4, moisture 74.3, and ash 1.3. Chicken meat showed the following mean composition: protein 23.6, total fat 2.5, moisture 72.6, and ash 1.3. The total caloric intake was 669, 521 and 536 Kcal for the pork, rabbit, and chicken meals, respectively.

### 2.2. Serum Collection

Fasting serum (FS) was obtained after an overnight fast of at least 10 h. Blood was withdrawn in the morning in order to obtain samples of fasting serum. Blood samples were collected into 7 ml Vacutainer serum tubes and placed 1 h at room temperature. After centrifugation, the serum was collected, divided into aliquots, and immediately frozen at −80°C. Fasting serum was pooled following the same strategy used for postprandial serum.

Postprandial serum was collected 4 hours after the meal from each volunteer. In order to ensure an adequate amount of postprandial serum for the experiments, adult volunteers consumed 5 meals of each type (for a total of 30 postprandial serum samples from each type of meal) and elderly volunteers consumed 2 meals of each type (for a total of 12 postprandial serum samples from each type of meal). After the establishment of individual serum lipid profiles, serum samples obtained from each meal were pooled (separately for adult and elderly volunteers) in order to dispose of 6 (elderly) or 10 (adult) pooled samples to be used in cell experiments (as described in Scheme 1 of the Supplementary Material).

### 2.3. Cell Culture and Proliferation

HCAECs (human coronary artery endothelial cells) were purchased from Clonetics Corporation (Lonza) and cultured in endothelial basal medium EBM, supplemented with EGM-2 or EGM-2/MV SingleQuots containing 0.1% rh-EGF (human recombinant epidermal growth factor), 0.04% hydrocortisone, 0.1% VEGF (vascular endothelial growth factor), 0.4% rh-FGF-B (human recombinant fibroblast growth factor), 0.1% rh-IGF-1 (insulin- like growth factor), 0.1% ascorbic acid, 0.1% heparin, 0.1% GA-1000 (gentamicin sulfate plus amphotericin B), and 10% fetal calf serum or 10% human fasting as well as postprandial serum.

HCAECs were plated at a seeding density of 2.500 cells/cm^2^ in T25 flasks or in 12-well cell culture plate in the presence of a medium containing 10% fetal calf serum or in presence of 10% human serum (fasting or postprandial). Cell cultures in the flasks reached confluence after 6-7 days, as assessed by light microscopic examination, and they were passaged at weekly intervals. Cell cultures in the well culture plate reached confluence after 3-4 days and were passaged two times per week. After trypsinization and before replating, harvested cells were counted using a haemocytometer and the number of population doublings (PD) were calculated using the following formula: (log10*F* − log10*I*) × 3.32 (where *F* indicates the number of cells at the end of the passage and *I* the number of cells when seeded) [17]. Endothelial cell senescence was studied by subjecting endothelial cells to subsequent passages until two consecutive population doublings equal or below 0 associated with senescence morphological changes revealed by microscopy examination. Cumulative population doubling (CPD) was calculated as the sum of all the changes in PD.

### 2.4. RNA Extraction

Total RNA was extracted from HCAECs subjected to the different treatments using the RNeasy Mini Kit (QIAGEN, Hilden, Germany) according to the manufacturer's protocol. Subsequently, RNA concentrations were assessed by NanoDrop spectrophotometer (260/280 absorbance ratios) and RNA quality was determined by gel electrophoresis.

### 2.5. Cellular Senescence Biomarkers

Senescence-associated beta-galactosidase activity (SA-beta-gal) was performed by flow cytometry as previously described [18]. Briefly, C12FDG was added to the pH modulation/buffer. HCAECs were incubated with this solution for 1 h at 37°C at 5% CO_2_. After incubation, HCAECs were trypsinized, washed with PBS, resuspended in 200 *μ*l PBS, and analyzed using a Coulter Epics XL flow cytometer. Data were analyzed with the instrument software, and cell debris was excluded on basis of light scatter parameters. C_12_FDG was measured on the FL1 median fluorescence intensity (MFI) of the HCAEC population.

For p16 expression, cDNA synthesis from total RNA was performed using the iScript reverse transcriptase (Bio-Rad, Hercules, CA) according to the manufacturer's guidelines. Messenger RNA for the housekeeping gene *β*-actin and for p16 was then measured by real-time PCR on a Bio-Rad iQ5 optical real-time thermal cycler (Bio-Rad, Hercules, CA) using 1 *μ*g of cDNA in a total volume of 20 *μ*l containing iQ SYBR Green Supermix (Bio-Rad, Hercules, CA). *β*-Actin was used as the reference gene. Forward and reverse primers used in this model have been previously reported [19]. Any inefficiencies in RNA input or reverse transcription were corrected by normalization to the housekeeping gene. Relative amounts of p16 were calculated using the threshold cycle (Ct) values obtained from qPCR and applying the ∆∆Ct method (ΔCt = Ct beta-actin − Ct p16 mRNA). A lower ΔCt value referred to lower expression of the p16 mRNA.

#### 2.6. Determination of Total Reactive Oxygen Species Production

Total cellular reactive oxygen species (ROS) production in HCAECs was analyzed by flow cytometry after loading of cells with a highly sensitive fluorescent probe, [5 and 6 chloromethyl-2′,7′-dichlorodihydrofluorescein diacetate, acetyl ester (CM-H2DCFDA)] (Molecular Probe, Life Technologies). One aliquot of 200, 000 cells was used as positive control and it was preincubated for 5 minutes with tert-butyl hydroperoxide solution (70%) diluted 1 : 100 in PBS then washed 2 times with PBS. All the other aliquots of HCAECs, including the positive control, were incubated at 37°C for 30 min in the dark with 2 *μ*M of probe in PBS buffer. CM-H2DCFDA is cleaved by intracellular esterases and transformed into a fluorescent dye when oxidized. Cells were then washed 2 times with PBS and analyzed by flow cytometry (Coulter Epics XL). The mean fluorescence intensity of 5, 000 cells (corrected for autofluorescence) was taken as a measure for the total ROS load.

#### 2.7. Measurements of Oxidative DNA Damage

HCAECs after 4 hours of exposure with postprandial serum pellets were trypsinized and one aliquot of 350.000 cells was frozen in liquid nitrogen then stored at −80°C. Another aliquot of 350.000 cells was plated in T25 flasks and incubated for 18 h at 37°C in the presence of a medium containing 10% fetal calf serum. The day after, the HCAECs were detached by trypsinization, then frozen in liquid nitrogen, and stored at −80°C until use. Genomic DNA was purified using the QIAamp DNA Micro Kit (Qiagen, Italy) following the manufacturer's protocol. Then, DNA was digested at 50° for 1 h with nuclease P1 (Sigma-Aldrich, Italy) following the manufacturer's instruction. After incubation with 1 unit of alkaline phosphatase at 37°C for 30 minutes, DNA samples were boiled for 10 minutes and placed in ice and subsequently stored at −20°C until use. 8-OHdG was measured using a competitive EIA assay (StressMarq Bioscience Inc.). The test utilizes an anti-mouse IgG-coated plate and a tracer consisting of an 8-OHdG-enzyme conjugate. This format has the advantage of providing low variability and increased sensitivity compared with assays that utilize an antigen-coated plate. Standard 8-OHdG was assayed over a concentration range of 0 to 3000 pg/ml in duplicates. Sample DNA assays were performed in duplicate, and the average concentration of 8-OHdG was expressed per nanogram of DNA.

### 2.8. Microarray Gene Expression Assays

The microarray procedure was performed according to the Affymetrix protocols (Santa Clara, CA, USA). In brief, 100 ng of total RNA was amplified and labeled using the GeneChip® 3′ IVT Plus kit (Affymetrix, Santa Clara, CA, USA). Hybridization cocktails containing fragmented, end-labeled single-stranded cDNA were prepared and hybridized to the GeneChip Human Genome U133 Plus 2.0 Array for 16 h at 45°C. (Affymetrix, Santa Clara, CA, USA). The signal intensities were measured using a GeneChip Scanner 3000 7G (Affymetrix) and converted to numerical data using the GeneChip Command Console® Software (AGCC). The microarray data analysis was performed by Partek® Genomics Suite 6.6 (Partek Inc., St Louis, MO, USA) using the default Partek normalization parameters. Affymetrix CEL files were imported and background correction and normalization were performed using GC-robust multiarray average (GC-RMA) algorithm. Comparisons among treatment groups were performed with the ANOVA tool implemented in the Partek software after removal of batch effects. In order to correct for multiple comparisons and reduce the number of false positives, a false discovery rate (FDR) adjusted *p* value <0.05 was used in combination with a fold change (FC) ≥ 2. Biofunctional analysis was performed using ConsensusPathDB (http://cpdb.molgen.mpg.de) [20] and Partek Pathway software (Partek, St Louis, MO, USA).

### 2.9. Statistical Analysis

Data were initially tested for normal distribution. Generalized linear models (for longitudinal data with normal distributed variables) or Kruskal-Wallis test (for nonnormal distributed variables) were used to compare data among experimental groups. Normal data were graphically represented as mean ± SEM while nonnormal data were represented as box plots. SPSS v. 23 was used as statistical software.

## 3. Results

### 3.1. Serum Triglyceride Levels in Postprandial Sera after Meat Meals

Baseline glucose, total cholesterol, HDL cholesterol, LDL cholesterol, and triglycerides were within the normal (70–110; <200; <100; >40; <150 for glucose, total cholesterol, HDL cholesterol, LDL cholesterol, and triglycerides) or borderline range (200–239; 130–159 for total and LDL cholesterol) in all subjects. As compared to fasting values, serum triglyceride levels and lipemic index increased 4 h after the meat meals (*p* < 0.01 versus baseline), while serum glucose was slightly decreased in sera from the elderly volunteers (Table 1). All the other variables remained unchanged. Lipemic index after the meal based on pork or rabbit was higher in elderly compared to adult volunteers (*p* < 0.05). The total amount of triglycerides was increased in the postprandial sera. The fatty acid composition of postprandial serum was only slightly different from the fasting serum composition. In fact, the postprandial serum contained few amount of some long chain fatty acids, docosatetraenoic (C22:4), lignoceric (C24:0), and nervonic (C24:1) acids, which were not detected in fasting serum (Supplementary Table 1).

### 3.2. Postprandial Sera Increase HCAEC ROS Production but Not Oxidative DNA Damage (8-OHdG) after a Few Hours of Exposure

**Figure 1:**
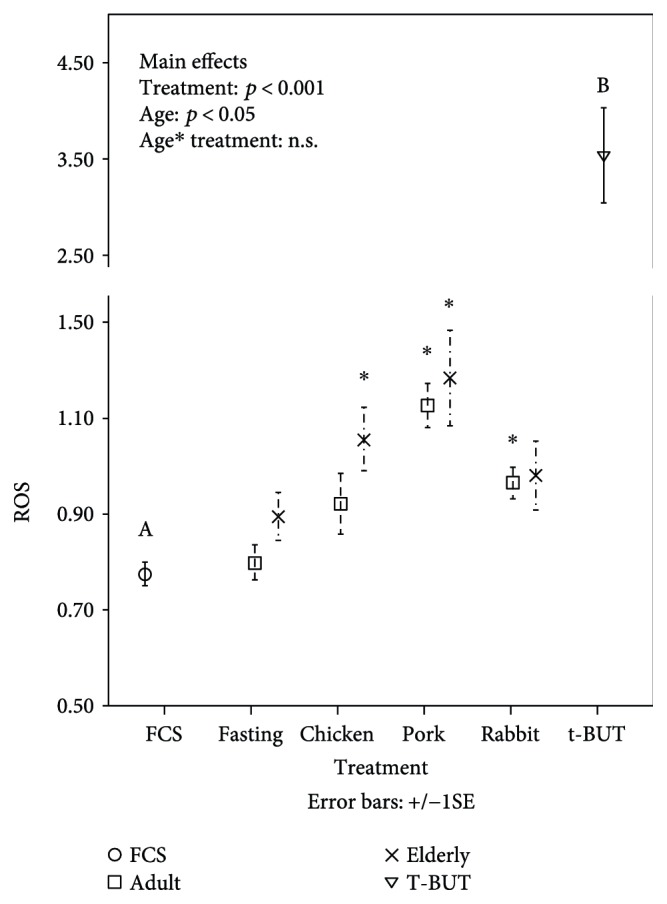
Effect of postprandial serum from adult (□) and elderly volunteers (×) after consumption of chicken, pork, or rabbit meat meals on HCAEC ROS production. Data are shown as mean ± SEM. All data are compared to fasting serum, fetal calf serum (FCS, marked by “○”) and the strong oxidant tert-butyl hydroperoxide (t-BUT, marked by “∇”). In the left-high panel are reported the significance of the main effects: age, treatment (postprandial versus fasting), and their interaction analyzed by generalized linear models. All other comparisons are derived from posthoc by LSD: ^∗^*p* < 0.05 versus the respective fasting serum, ^A^*p* < 0.01 versus all postprandial sera (chicken, pork, and rabbit), and ^B^*p* < 0.01 versus all other treatments. Pooled samples used for the experiments were *n* = 6 (elderly) and *n* = 10 (adult). Each sample was run as a technical triplicate. For control conditions, *n* = 10 (FCS) and *n* = 6 (t-BUT).

**Figure 2:**
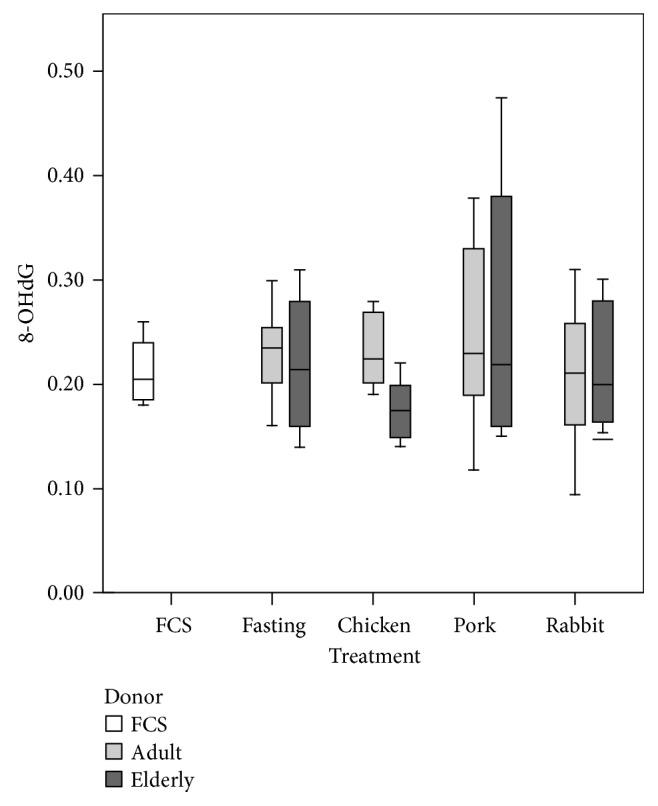
Effect of postprandial serum from adult and elderly volunteers after consumption of chicken, pork, or rabbit meat meals on HCAECs oxidative DNA damage (8-OHdG). Data are shown as box plots representing the median, interquartile range, and top and bottom whiskers (highest and lowest case within 1.5 times IQR). No significant differences were observed by the Kruskal-Wallis test or ANOVA even when data of postprandial sera are considered together and compared to fasting sera. Samples used for the experiments were *n* = 6 (elderly) and *n* = 10 (adult). Each sample was run as a technical duplicate. For control condition in fetal calf serum (FCS), *n* = 4.

Incubation (4 h) with serum collected 4 h after the pork meat lunch resulted in a significant increase in HCAEC ROS compared to incubation with preprandial (fasting) serum (Figure 1). Conversely, no difference in ROS was detected between HCAEC grown in their own media versus HCAEC incubated with FS (Figure 1). The levels of ROS induced by incubation with postprandial sera were consistently lower than those induced by treatment with t-But (Figure 1). We detected no significant changes in 8-OHdG in HCAEC exposed 4 h to the postprandial serum compared to fasting serum (Figure 2), neither there was any significant difference after replacement of HCAEC in their own medium for 18 h.

### 3.3. Transcriptional Profiling of HCAEC Cells Exposed to Fasting or Postprandial Serum from Adult and Old Donors

**Figure 3:**
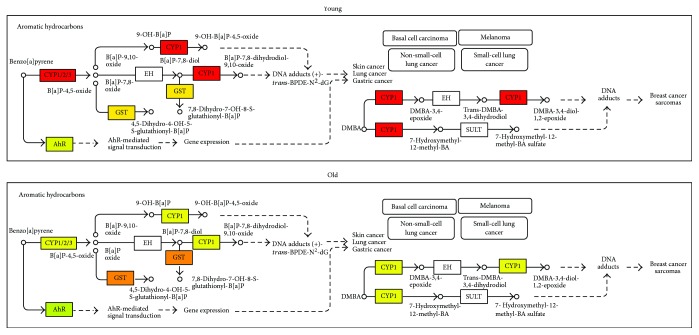
Aromatic hydrocarbon pathway and related significant changes observed in response to postprandial serum (red: strongly upregulated compared to fasting serum; orange: mildly upregulated compared to fasting serum; green: downregulated compared to fasting serum).

**Figure 4:**
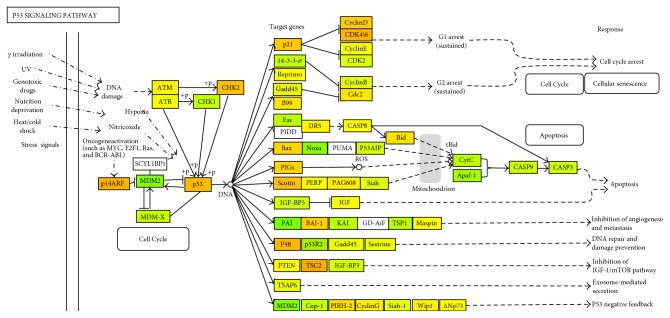
p53 signaling pathway and related significant changes observed in response to postprandial serum (orange: upregulated compared to fasting serum; green: downregulated compared to fasting serum).

**Figure 5:**
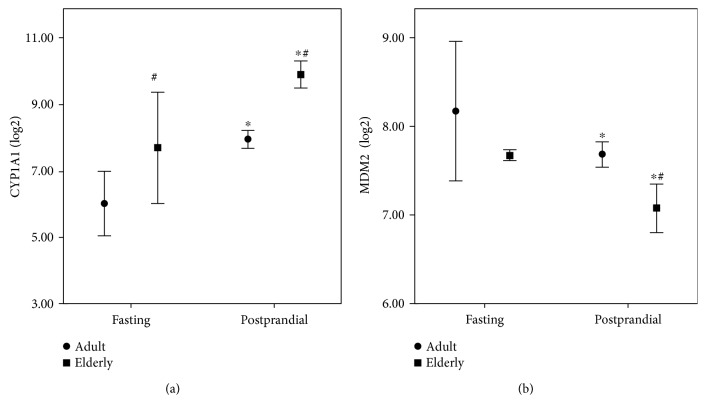
Log2 intensity of CYP1A1 and MDM2 genes in HCAEC exposed to fasting or postprandial serum from adult and elderly donors. ^∗^*p* < 0.01 compared to fasting serum; ^#^*p* < 0.01 compared to adult donors.

Global changes in gene expression were analyzed in HCAEC cells treated for 4 h with fasting or postprandial sera withdrawn from volunteers after the pork meat meals, which showed the highest prooxidative impact on HCAEC (Figure 1). A minimum of 4 independent replicates was performed for each condition. Two-way ANOVA identified 2877 genes significantly modulated by postprandial serum versus fasting serum (*p* < 0.05). Of these genes, 51 were significantly expressed (*p* < 0.05 and FDR < 0.05) with absolute fold change > 1.2. The top 20 genes with the greatest fold change showing the same direction in HCAEC exposed to elderly and adult postprandial serum are listed in Table 2. The most affected pathway was searched including the whole gene list in the gene set analysis tool available by ConsensusPathDB (http://cpdb.molgen.mpg.de) [20]. Significant pathways that were useful to the human endothelial cellular model are displayed in Table 3. The results confirm that the most important biological function affected by postprandial serum exposure include detoxification (chemical carcinogenesis, xenobiotic metabolism, and drug metabolism), oxidative stress (oxidative stress-induced senescence, MAPK signaling pathway, and oxidative stress), cellular senescence (oxidative stress-induced senescence and cellular senescence), and mediators of inflammation (TNFR1-induced NF-*κ*B signaling pathway and TNF signaling). Two representative pictures including those of the most important players involved in detoxification (aromatic hydrocarbons pathway) and oxidative stress-induced senescence (p53-pathway) were drawn by Partek Pathway (Figures 3 and 4). CYP1A1 (an important player in detoxification function) and MDM2 (an important player in p53-signalling and oxidative stress response) were the two genes represented in most pathways and were additionally characterized by marked fold changes (Figure 5). Additionally, CYP1A1 was significantly higher in cells exposed to elderly donors' serum (both fasting and postprandial) compared to adult donors' serum, whereas MDM2 was lower in cells exposed to elderly donors' postprandial serum compared to adult donors' postprandial serum.

### 3.4. Repeated Exposure to Postprandial Sera Slightly Accelerates Cellular Senescence in HCAEC Compared to Fasting Sera

**Figure 6:**
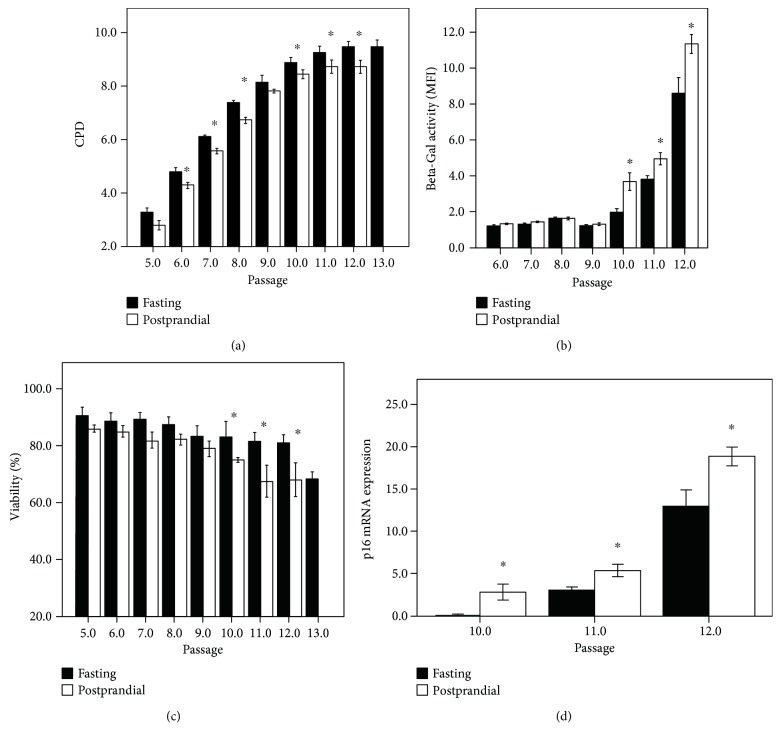
The effect of culture with postprandial serum (2 times per day for 4 h) compared to fasting serum (2 times per day for 4 h) on growth and senescence of HCAECs. Postprandial serum used for these experiments was obtained from adult volunteers after the pork meat meals. All data are mean ± SEM for *n* = 4–6 independent cell populations for each condition (fasting serum and postprandial). (a) Cumulative population doubling (CPD) was calculated using the formula 3.32^∗^(log10*F* − log10*I*) over consecutive population doublings. (b) Senescence-associated *β*-galactosidase (SA-*β*-gal) activity for cells grouped by passage number and treatment as indicated. (c) Viability for cells grouped by passage number and treatment as indicated. (d) p16 expression after passage 9 for cells grouped by passage number and treatment as indicated. Data are means with error bars showing SEM. ^∗^*p* < 0.05 compared to fasting serum.

HCAEC cultured in wells and in flasks in normal medium showed the phenotypic appearance of senescence after 4–6 weeks (10–12 CPD) and 6–8 weeks (18–20 CPD), respectively. After this period, the cells flattened became irregular in shape with an increased diameter, thus exhibiting the characteristic of senescent cells including beta-Gal staining. Although the “well system” was shown to accelerate the appearance of the senescence features and to reduce the proliferative potential in HCAEC, we were forced to adopt this system for the comparison of the effects of repeated exposure to fasting and postprandial sera due to the limited availability of the biomaterial (in particular, human postprandial serum). In the first set of experiments (performed in the “flask system”), we investigated if a single exposure (4 h) of early passage HCAEC to postprandial serum from different meat meals could have an impact on growth curve and senescence of the cells. In these experiments, it did not find any impact of postprandial serum on growth curves (measured as CPDs at each passage) of HCAEC (Supplementary Figure 1). In order to study the impact of repeated exposure to postprandial serum on replicative senescence of HCAEC, we used the “well system” and the postprandial serum obtained from adult volunteers after the pork meat meals, as this serum induced the highest oxidative stress after a single exposure in HCAECs (Figure 1). When fasting serum was added two times a day for 4 h, no distinct and reproducible differences in CPD or SA-beta-gal activity were observed between treated and untreated cultures (the medium of untreated cultures was replaced with the same timing and procedure for treated cells) during the whole growth curve (data not shown). Conversely, a reduction of CPDs compared to HCAEC treated with fasting serum was observed in HCAEC treated with postprandial serum added two times a day (for 4 h) from early passages up to the onset of replicative senescence (Figure 6). Moreover, when compared to the fasting serum, SA-beta-gal activity was higher in the late passages of HCAEC treated two times a day with postprandial serum starting from early passages up to the onset of replicative senescence (Figure 6). We observed also a loss of viability and an increased expression of p16 after passage 10 in cells exposed to postprandial serum compared to treatment with the fasting serum (Figure 6).

## 4. Discussion

This study aimed to answer the question whether acute and chronic exposure of HCAECs to postprandial serum withdrawn from volunteers after a normal meal composed of oven-cooked meat with seasoning and side dishes can have detrimental effects on endothelial cells and if these effects are influenced by the age of the donors. We found that exposure of HCAECs to postprandial serum, withdrawn independently from adult and elderly individuals, can induce a slight increase in ROS production. The postprandial serum-induced oxidative stress is also associated with the expression of genes involved in oxidative stress response, detoxification from chemical compounds, cellular senescence, and inflammation. However, we did not find any evidence of DNA damage; thus, suggesting that a single exposure of cells to postprandial serum is compatible with a mild stress event. Indeed, once the postprandial serum is removed and the cells are regrown in normal condition, the proliferative activity and the onset of replicative senescence is not affected. Conversely, repeated exposure of HCAEC to postprandial serum (2 times a day) over time affects the proliferative activity and accelerates the onset of replicative senescence *in vitro*. Taking into account that the accumulation of endothelial senescent cells is a common mechanism at the basis of atherosclerosis [21–23] and other age-related diseases [24], these data could form the rationale to explore a new mechanistic link among dietary habits and risk of age-related diseases.

Consistent with previous studies performed by administration of high-fat challenges in healthy men [9, 25, 26], we found that triglycerides and lipemic index increase in plasma 4 h after the oven-cooked meat meal (including potatoes on the side, seasoning, and half glass of wine). We additionally observed that lipemic index after the pork or rabbit meat meal were higher in elderly compared to adult volunteers. As documented previously, the magnitude of postprandial lipemia is increased with aging [27, 28], a phenomenon that might place elderly individuals at greater risk for cardiovascular disease compared with their adult counterparts. This phenomenon likely occurs as a consequence of a dysregulated secretion of hepatic triglycerides into the plasma during the postprandial period [28]. Postprandial lipemia is also associated with a general metabolic stress, characterized by the rise in oxidative stress and proinflammatory cytokines as well as endotoxemia which has been reported to be similar in younger adults and healthy older adults [29]. Indeed, in spite of the higher lipemic index in postprandial sera from the elderly, we observed that the increase in oxidative stress of HCAECs exposed to postprandial sera was comparable between sera derived both from elderly and adult volunteers, albeit the ROS were in general higher with sera derived from the former. In any case, it is possible that prooxidant mediators are already present in serum after meal consumption [29]. Alternatively, another hypothesis is that the high burden of lipids forces mitochondrial oxidation with consequent production of ROS in analogy to the phenomenon described in response to hyperglycemia [30]. This could eventually explain the proportionality of the results between the lipemic index with the various kinds of meals and the level of ROS.

It has been previously shown that human umbilical vein endothelial cells (HUVECs) exposed to sera withdrawn 4 h after a fat challenge decrease their proliferative activity [9]. However, evidence is increasing that HUVECs, derived from a vascular bed not present in the adult, differ phenotypically and epigenetically from adult cells, such as HCAECs [31]. Although our volunteers ingested less than half of the fat usually administered during a dietary fat challenge (approx. 50 g), we also detected a slight decrease in the spontaneous proliferation of the HCAECs repeatedly exposed to postprandial sera, which is consistent with the observed increase in oxidative stress. This decrease in proliferative activity might be interpreted as a compensatory and beneficial effect to avoid additional damage in the presence of stress. Indeed, a single exposure (4 h) of HCAECs to postprandial serum induces only a transient and modest increase in oxidative stress with the activation of several defensive pathways (detoxification pathways, oxidative stress response, and p53) that are reversed when cells are regrown in their normal medium (data not shown) without functional impairment (as demonstrated by the normal growth curve and replicative senescence timing). Conversely, when the exposure is repeated two times a day for the whole cellular lifespan, the impact of postprandial serum on the growth and senescence of the HCAEC is revealed. Subtoxic oxidative stress in cell culture is known to generate a transient adaptive response which may eventually produce a beneficial hormetic effect [32]. However, in the case of chronically repeated exposure to an excess of nutrients, the adaptive response might eventually culminate in a proliferative arrest and cellular senescence [33]. The process may be similar to the one reported for the acceleration of senescence induced in cultured human cells by high concentrations of glucose in the medium [34, 35]. Our transcriptomic results and pathway analysis showed an additional hypothesis. Indeed, postprandial serum promotes the activation of pathways involved in the detoxification by chemical carcinogens, in particular, the aromatic hydrocarbons detoxification pathway. This is well represented by the postprandial-induced expression of CYP1A1 (Figures 3 and 5). This gene (a member of the superfamily of enzymes of cytochrome P450) is not only involved in metabolizing a multitude of polycyclic aromatic hydrocarbons and other chemical compounds (including carcinogens) that arise from meat cooking [36] but also in the conversion of polyunsaturated fatty acids into signaling molecules that have physiological as well as pathological activities. Dietary fatty acids can mediate P450 induction [37] and are agonists of Toll-like receptors, which can explain the modulation of inflammatory signature after exposure to postprandial sera and the altered expression of CYP1A1 [38]. Interestingly, the basal levels of CYP1A1 were upregulated in the cells exposed to the elderly's postprandial serum compared to the adult one, which may be consistent in the increase in serum free fatty acids with aging [39]. Regarding the production of potential carcinogens, we know that red and white meats cooked at high temperature may have high levels of meat mutagens, including benzo[a]pyrene [40], one of the most studied polycyclic aromatic hydrocarbons derived from cooked meat. Since exposure to polycyclic aromatic hydrocarbons has been implicated not only in colorectal cancer [41] but also in cardiovascular diseases [42], it might formed a speculative hypothesis on the possible influence of these compounds on endothelial senescence. By the way, benzo[a]pyrene was found to restrain cell cycle in murine brachial epithelial cells [43], to increase the mutation frequencies and carcinogenesis *in vivo* in the lacZ transgenic (mutation reporter) mouse [39] and to induce p53 protein accumulation, a mediator of cellular senescence, in various cellular models [44, 45]. This last result is consistent with our finding of an upregulation of cellular senescence and p53 pathway in the HCAEC model exposed to postprandial serum. Our analysis additionally suggests that NF1 downregulation is induced in HCAEC exposed to postprandial serum. This could be relevant in the acceleration of replicative senescence as NF1 loss is known to induce senescence in human melanocytes [46] and fibroblasts [47] as well as altered vascular morphogenesis in human endothelial cells [48]. Last but not least, our pathway analysis reported that TNF and NF-*κ*B signaling are affected by exposure to postprandial serum. While we have not been able to predict if this modulation can result in a pro- or anti-inflammatory response, the results are in agreement with the studies that have demonstrated that the postprandial phase is characterized by mediators of inflammation able to modulate NF-*κ*B and tissue expression of TNF-alpha during the postprandial phase [49, 50]. By the way, it has been recently demonstrated that treatment of peripheral blood mononuclear cells with postprandial VLDL lipolysis products increased expression of TNF*α*, IL-1*β*, and IL-8 over controls, with concurrent activation of NF-*κ*B [51].

At our knowledge, our study addresses for the first time the potential consequences of a repeated exposure to this specific environment. Unfortunately, it is not possible to disentangle the effects of the inflammatory mediators from oxidative stress as well from the potential exposure to chemicals arising from the cooking process. However, it is likely that all of them act synergically in the establishment of the functional outcome (reduced proliferative activity and acceleration of senescence following repeated exposure) of postprandial serum on endothelial cells. Interestingly, a very similar *in vitro* technique, where cell lines have been exposed to the serum collected from humans or animals on calorie-restricted diets, has been proposed as a tool to predict the potential of dietary manipulations to affect markers of longevity [52, 53]. Indeed, cell cultures exposed to the postprandial serum from subjects at the end of a caloric restriction trial showed greater resistance to oxidative stress, upregulation of longevity genes, an increase of Sirt1, a proliferation reduction, and an increase in thermal shock resistance [52]. Considering that these experiments were developed on hepatic cancer cells that do not suffer from replicative senescence, our model of exposure by using human primary endothelial cells might be a useful advance. One of the limits of our model is that we cannot take into account the physiological fluctuation of the postprandial period depending on hormonal excretions and other factors that characterize the *in vivo* condition. Moreover, the clinical markers of the elderly enrolled in this study were consistent with an exceptional health status. This fact may eventually hamper the generalization of the findings to the elderly population.

On the basis of these results, it is possible to conclude that treating *in vitro* endothelial cells with postprandial sera could be a useful experimental tool to monitor the potential health impact of dietary intervention and challenges. This experimental tool could be useful in the design of experiments aimed to study the impact of processed foods on health, especially in the context of Western societies, where a significant part of the day is spent in the postprandial state [6].

## Figures and Tables

**Table 1:** Changes in the serum glucose, total cholesterol, HDL cholesterol, LDL cholesterol, triglyceride levels, and lipemic index after 4 h from the meat meal consumption. Data represent mean ± SEM.

	FS		CMS		RMS		PMS	
	Adult	Elderly	Adult	Elderly	Adult	Elderly	Adult	Elderly
Glucose (mg/dl)	91 ± 9	97 ± 9	87 ± 14	78 ± 5^∗∗^	87 ± 8	83 ± 5^∗^	90 ± 12	85 ± 3^∗^
Lipemic index (mg/dl)	12 ± 5	10 ± 2	44 ± 17^∗^	45 ± 15^∗^	50 ± 20^∗#^	74 ± 43^∗∗^	98 ± 49^∗∗#^	154 ± 64^∗∗^
Triglycerides (mg/dl)	86 ± 25	82 ± 29	158 ± 27^∗∗^	165 ± 19^∗∗^	167 ± 29^∗∗^	170 ± 39^∗∗^	185 ± 63^∗∗^	215 ± 47^∗∗^
HDL cholesterol (mg/dl)	59 ± 14	68 ± 15	55 ± 11	60 ± 13	50 ± 11	64 ± 7	53 ± 10	62 ± 7
LDL cholesterol (mg/dl)	133 ± 31	106 ± 26	126 ± 36	105 ± 29	112 ± 32	87 ± 17	117 ± 25	84 ± 17
Total cholesterol (mg/dl)	204 ± 33	190 ± 27	199 ± 43	189 ± 22	176 ± 43	176 ± 13	196 ± 29	188 ± 16

^∗^ *p* < 0.05 versus the respective FS; ^∗∗^*p* < 0.001 versus the respective FS; ^#^*p* < 0.05 versus elderly group. FS: fasting serum; CMS: chicken meal serum; RMS: rabbit meal serum; PMS: pork meat serum.

**Table 2:** Representative genes with greatest fold differences in HCAEC exposed to fasting or postprandial^∗^ serum from adult and elderly donors.

Probeset ID	Gene symbol	Gene name	Fold-change postprandial versus fasting adult serum	*p* value	Fold-change postprandial versus fasting elderly serum	*p* value
*(a) Upregulated genes*						
205749_at	CYP1A1	Cytochrome P450, family 1, subfamily A	3.7992	<0.0001	2.4956	0.0008
228770_at	GPR146	G protein-coupled receptor 146	2.0300	0.0003	1.8857	0.0361
227652_at	FAM69B	Family with sequence similarity 69, member B	1.5366	0.0264	1.9603	0.0330
221565_s_at	CALHM2	Calcium homeostasis modulator 2	1.4354	0.0143	2.3629	0.0006
209830_s_at	SLC9A3R2	Solute carrier family 9, subfamily A	1.3971	0.0364	3.4436	<0.0001
219020_at	HS1BP3	HCLS1 binding protein 3	1.3794	0.0026	1.5682	0.0089
226488_at	RCCD1	RCC1 domain containing 1	1.3371	0.0127	1.7111	0.0054
211143_x_at	NR4A1	Nuclear receptor subfamily 4, group A, member 1	1.2800	0.0264	1.5553	0.0159
223415_at	RPP25	Ribonuclease P/MRP 25 kDa subunit	1.2674	0.0352	1.8069	0.0020
207978_s_at	NR4A3	Nuclear receptor subfamily 4, group A, member 3	1.2430	0.0310	1.4449	0.0261
*(b) Downregulated genes*						
210631_at	NF1	Neurofibromin 1	−1.6760	0.0017	−1.8129	0.0228
1569867_at	EME2	Essential meiotic structure-specific endonuclease	−1.4108	0.0338	−1.7198	0.0406
225160_x_at	MDM2	MDM2 protooncogene, E3 ubiquitin protein ligase	−1.4035	0.0112	−1.5440	0.0435
1553113_s_at	CDK8	Cyclin-dependent kinase 8	−1.3286	0.0020	−1.6654	0.0008
224712_x_at	SMIM7	Small integral membrane protein 7	−1.3229	<0.0001	−1.3019	0.0067
1552487_a_at	BNC1	Basonuclin 1	−1.3185	0.0470	−1.6384	0.0311
226881_at	GRPEL2	GrpE-like 2, mitochondrial (*E. coli*)	−1.3133	0.0129	−1.4588	0.0332
229027_at	PPM1A	Protein phosphatase, Mg2+/Mn2+ dependent, 1A	−1.3019	0.0236	−1.5777	0.0173
228397_at	TUG1	Taurine upregulated 1 (nonprotein coding)	−1.2723	0.0400	−1.5252	0.0285
221203_s_at	YEATS2	YEATS domain containing 2	−1.2717	<0.0001	−1.3036	0.0024

^∗^The present analysis is restricted to the top 10 upregulated and downregulated genes expressed in HCAEC treated with postprandial sera withdrawn after pork meat meals versus fasting serum.

**Table 3:** Enriched pathway-based sets^∗^ identified by ConsensusPathDB.

Pathway name	Set size	Candidates contained	*p* value	*q* value	Pathway source
Chemical carcinogenesis	82	18 (22.0%)	0.000842	0.203	KEGG
MAPK signaling pathway	168	30 (17.9%)	0.000889	0.203	WikiPathways
Xenobiotics metabolism	53	13 (25.5%)	0.00101	0.203	EHMN
Drug metabolism	46	12 (26.1%)	0.00126	0.203	KEGG
TNFR1-induced NF-*κ*B signaling pathway	26	8 (30.8%)	0.00263	0.232	Reactome
Oxidative stress-induced senescence	129	23 (18%)	0.0031	0.242	Reactome
Ubiquitin-mediated proteolysis	137	24 (17.5%)	0.00359	0.243	KEGG
Cellular senescence	192	30 (15.7%)	0.00654	0.318	Reactome
Oxidative stress	30	8 (26.7)	0.00693	0.318	WikiPathways
The fatty acid cycling model	5	3 (60%)	0.00812	0.318	Reactome
TNF signaling	37	9 (24.3%)	0.00816	0.318	Reactome

^∗^1243 genes (59.7%) from the input list (2877 genes) are present in at least one pathway.

## Supplementary Materials

Supplementary materials Scheme 1: Design of the pooling strategy adopted with the withdrawal obtained after each meal in order to get the samples used for the experiments with the HCAECs “in vitro.” Supplementary Table 1: Fatty acid composition^∗^ of pork meat, fasting serum and postprandial serum after consumption of a meal based on pork meat. Supplementary Figure 1. Growth curves of HCAECs cultured in flasks after a single exposure (4H) to various postprandial sera. No difference was reported in comparison to treatment with fasting serum and to normal culture conditions with fetal calf serum (not shown in this figure).
