# Supplementary material for: Gene Expression, Oxidative Stress, and Senescence of Primary Coronary Endothelial Cells Exposed to Postprandial Serum of Healthy Adult and Elderly Volunteers after Oven-Cooked Meat Meals
Source: Mediators Inflamm. 2017 Dec 12;2017:3868545. doi: 10.1155/2017/3868545 (PMC5742900; doi:10.1155/2017/3868545)

**Supplementary Data**

**Scheme 1. Design of the pooling strategy adopted with the withdrawal obtained after each meal in order to get the samples used for the experiments with the HCAECs ”in vitro”.**

**
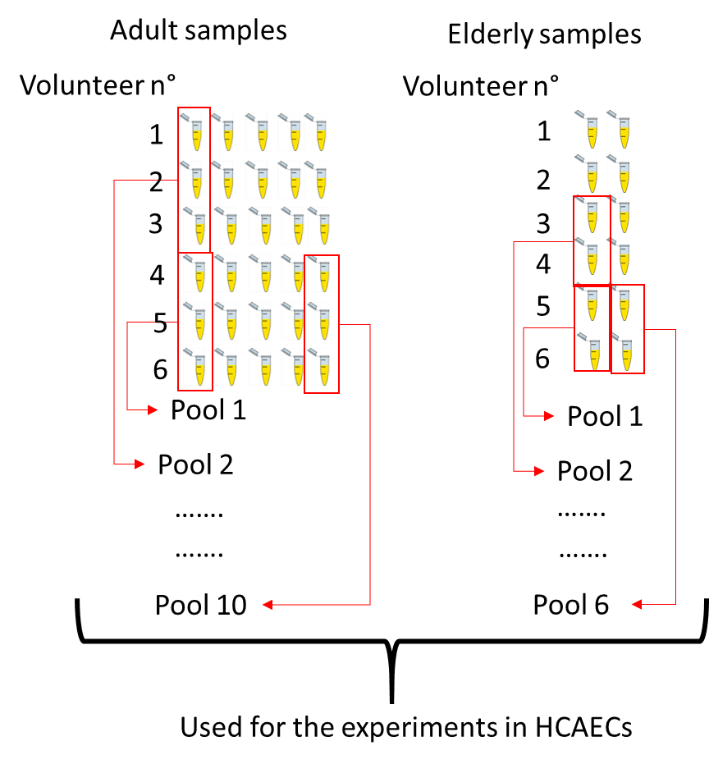
**

**Determination of fatty acids composition in pork meat**

*Sampling and Extraction of Total Lipids from pork meat*

The pork loins were sliced to obtain two chops of about 200 g of weight, which were packed and stored at -18°C for few days until fat extraction. Four fresh pork loins (*Longissimus lumborum*) were randomly collected (two from male and two from female pigs) and utilized for lipid extraction. An aliquot (20 g) of each muscle tissue was homogenized in chloroform − methanol (1:2, v/v) and the lipids were extracted according to Bligh and Dyer method.

*Analysis of Fatty Acid Profile.*

Fatty acid methyl esters (FAMEs) were obtained from total lipids through alkaline transmethylation. The qualitative analysis of FAMEs was carried out using a Focus gas chromatograph (Thermo Electron Corporation, West Palm Beach, FL, USA) equipped with a CP-Sil88 fused silica capillary column (100 m × 0.25 mm i.d., film thickness 0.2 μm, Chrompack, Middelburg, The Netherlands) and a quadrupole mass detector (FocusDSQ). The carrier gas was helium at a flow rate of 1.6 mL min^−1^; the oven temperature program started from 160 °C, raised to 240 °C at a rate of 4 °C min^−1^ and remained at 240 °C for 10 minutes. The injector temperature was 260 °C. The sample was injected into a split/splitless system. The ion source temperature of the mass detector was set at 260 °C. The mass spectrum was acquired using Xcalibur Data System ver. 1.4. Peaks were identified by comparison with known standards and using the NIST mass spectral database. The quantitative analysis of FAMEs was performed by means of gas chromatography using a CP-9002 apparatus (Chrompack, Middelburg, The Netherlands) equipped with a flame ionization detector (FID) and the same column and operative conditions reported above. The temperature of the detector was set at 260 °C. A Supelco (Bellefonte, PA, USA) standard solution containing a mixture of 37 FAMEs was used for identification of peaks and for the calculation of correction factor of the individual fatty acid peak areas. Fatty acid compositions (wt %) were calculated by the corrected peak area normalization method.

**Determination of fatty acids composition in the post-prandial serum**

*Transmethylation of serum fatty acids by boron trifluoride*

Transmethylation reaction is performed following the method described by Harris et al. with some slight modification. 100 µl of serum and 10 µl of internal standard solution (1.43 mg/ml nonadecanoic acid (Sigma Aldrich®) in toluene) are freeze-dried in a 2 ml screwed cap vial. 250 µl of a solution of BF3 (12% in MeOH anhydrous from Sigma Aldrich®) and 250 µl of hexane are added in sequence to the freeze-dried sample. The vial is capped and the suspension homogenized by vortexing for 15 seconds. Then, the vial is put in the oven at 100°C for 10 minutes. Once removed from the oven, the vial is suddenly cooled. 250 µl of H2O are added to the sample to completely stop the reaction and the mixture is vortexed for 20 seconds. Two phases are obtained after centrifugation. In the upper phase methyl esters from the fatty acids are dissolved in hexane. 150 µl of the upper phase are transferred in a 300 µl screwed cap vial. The solution is dried under nitrogen flow, and the methyl esters are resuspended in 50 µl of hexane to enable the GC-FID analysis.

*GC-FID analysis of methyl esters*

Varian® 430 GC is employed, equipped with a split/splitless injector, a 100m Varian® capillary column Select™ FAME (0.25 mm, 0.25 µm, #CP7421) and a flame ionization detector (FID).
2 µl of the methyl ester solution are injected with a split ratio of 2. The injector and the FID are maintained at 260°C. Carrying gas is He, 1.6 ml/min. The temperature gradient of the column start from 160°C till to 240°C in 20 minutes and remains at 240°C for 15 minutes.

Identification of FAMEs is attended comparing chromatograms from the samples and from analytical standard (FAME mix C4-C24 unsaturates, purchased from Sigma Aldrich ®). Peaks integration is performed by Galaxie software. Data are processed by Excel software.

**Suppl. Table 1. Fatty acid composition* of pork meat, fasting serum and postprandial serum after consumption of a meal based on pork meat**

| **Fatty acid (%)** | **Pork Meat** | **Fasting Serum** | **Postprandial Serum** |
| --- | --- | --- | --- |
| C12:0 | 0.1±0.0 | n.d. | n.d |
| C14:0 | 1.5±0.0 | 0.7±0.3 | 0.6±0.1 |
| C16:0 | 25.3±0.5 | 30.0±1.0 | 28.8±0.5 |
| C17:0 | 0.2±0.0 | 0.4±0.1 | 0.4±0.1 |
| C18:0 | 14.5±0.4 | 12.4±0.7 | 13.4±0.2 |
| C20:0 | 0.2±0.0 | n.d. | n.d. |
| **∑ SFA** | 41.7±0.4 | 43.5±0.9 | 43.4±0.8 |
| C16:1 Δ9c | 2.3±0.2 | 1.2±0.2 | 0.8±0.1 |
| C16:1*iso* | 0.3±0.1 | n.d | n.d |
| C18:1Δ^9^*^t^* | 0.1±0.0 | n.d. | n.d. |
| C18:1Δ^9c^ + C18:1Δ^11c^ | 42.7±0.5 | 17.4±1.1 | 16.5±1.9 |
| C20:1Δ^11c^ | 1.0±0.1 | 0.1±0.0 | 0.2±0.0 |
| **∑ MUFA** | 46.5±0.3 | 19.6±1.2 | 19.2±1.9 |
| C18:2 Δ^9t,12t^ | 0.1±0.0 | n.d | n.d |
| C18:2Δ^9c,12c^ [ω6] | 9.8±0.6 | 19.4±3.0 | 18.7±1.8 |
| C18:3Δ^9c,12c,15c^ [ω3] | 0.4±0.0 | 0.2±0.1 | 0.1±0.1 |
| C18:2Δ^9c,11 t^ [CLA] | 0.1±0.0 | n.d | n.d |
| C20:2Δ^11,14^ [ω6] | 0.5±0.0 | n.d | n.d |
| C20:3Δ^8c,11c,14c^ [ω6] | 0.1±0.0 | 2.3±0.4 | 2.0±0.3 |
| C20:4Δ^5c,8c,11c,14c^ [ω6] | 0.6±0.0 | 8.3±0.9 | 7.8±1.4 |
| C22:4Δ^7c,10c,13c,16c^ [ω6] | 0.1±0.1 | nd | 0.6±0.2 |
| C24:0 | nd | nd | 1.6±0.3 |
| C24:1 | nd | nd | 1.0±0.2 |
| **∑ PUFA** | 11.8±0.8 | 35.1±1.9 | 33.7±1.5 |
| **PUFA/SFA** | 0.3±0.0 | 0.8±0.1 | 0.8±0.0 |
| **PUFA ω3/PUFA ω6** | 0.1±0.0 | 0.2±0.1 | 0.2±0.1 |

*Fatty acid composition of pork meat (loins of *Longissimus lumborum* muscle) represent weight % of total fatty acids from 4 independent samples. Fasting serum and postprandial serum represent weight % of total fatty acids from 3 pooled samples from obtained young volunteers before and after the meal; n.d. = not detected. Data are displayed as mean ± SD.

**Suppl. Figure 1.** Growth curves of HCAECs cultured in flasks after a single exposure (4H) to various postprandial sera. No difference was reported in comparison to treatment with fasting serum and to normal culture conditions with fetal calf serum (not shown in this figure).


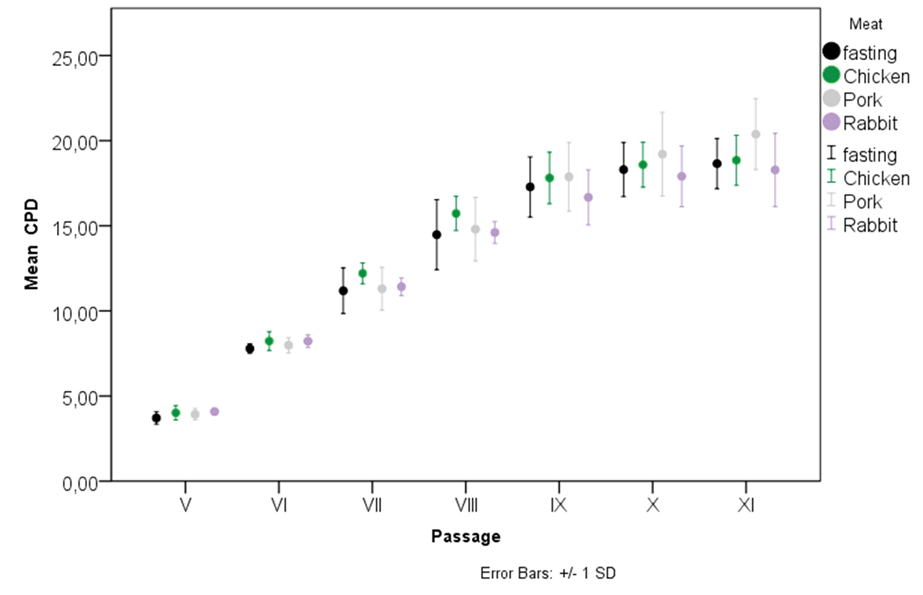

Supplement: Supplementary materials — Scheme 1: Design of the pooling strategy adopted with the withdrawal obtained after each meal in order to get the samples used for the experiments with the HCAECs “in vitro.” Supplementary Table 1: Fatty acid composition∗ of pork meat, fasting serum and postprandial serum after consumption of a meal based on pork meat. Supplementary Figure 1. Growth curves of HCAECs cultured in flasks after a single exposure (4H) to various postprandial sera. No difference was reported in comparison to treatment with fasting serum and to normal culture conditions with fetal calf serum (not shown in this figure). [file 3868545.f1.docx]
